# The Role of Nerve Exploration in Supracondylar Humerus Fracture in Children with Nerve Injury

**DOI:** 10.5704/MOJ.1511.019

**Published:** 2015-11

**Authors:** RIM Anuar, SG Gooi, O Zulkiflee

**Affiliations:** Department of Orthopaedics, Penang Hospital, Georgetown, Malaysia

**Keywords:** Nerve exploration, supracondylar humerus fracture, nerve injury

## Abstract

The supracondylar humerus fracture (SCHF) in children is common and can be complicated with nerve injury either primarily immediate post-trauma or secondarily posttreatment. The concept of neurapraxic nerve injury makes most surgeons choose to ‘watch and see’ the nerve recovery before deciding second surgery if the nerve does not recover. We report three cases of nerve injury in SCHF, all of which underwent nerve exploration for different reasons. Early reduction in the Casualty is important to release the nerve tension before transferring the patient to the operation room. If close reduction fails, we proceed to explore the nerve together with open reduction of the fracture. In iatrogenic nerve injury, we recommend nerve exploration to determine the surgical procedure that is causing the injury. Primary nerve exploration will allow early assessment of the injured nerve and minimize subsequent surgery.

## Introduction

The supracondylar humerus fracture (SCHF) is one of the most common fractures in children, predominantly the extension-type. As the Median and Radial Nerves lie anterior to the supracondylar humerus region they are at risk for injury, primarily post-trauma either by stretching, piercing or impinging at the fracture ends or being entrapped between two fracture fragments (traumatic or primary nerve injury), whereas the Ulnar Nerve injury is usually secondary to treatment (iatrogenic or secondary nerve injury)^1^.

The documented incidence of primary nerve injury in SCHF is 7 – 10% and up to 6% for secondary nerve injury^1^. In the extension-type of SCHF, postero-medial displacement of distal fragment usually causes injury to the Radial Nerve and postero-lateral placement is more likely to cause Median Nerve injury. In the flexion-type of SCHF, the Ulnar Nerve injury predominates^2^. Closed manipulative reduction (CMR) and percutaneous pinning are the first line treatments for SCHF^3^. Various methods of pin placement have been described and crossed pinning is still popular among surgeons. For the medial placement of the Kirschner-wire (K wire), the mini-open technique has been proposed in order to minimize injury to the Ulnar Nerve. In lateral pinning, the Median Nerve is at risk of injury^2^.

The concept of traumatic neurapraxia and iatrogenic neurapraxia are well understood and accepted by most of surgeons, but some orthopaedic surgeons still believe in acute nerve decompression or nerve exploration as these have their own benefits. We discuss three cases of nerve injury in SCHF which underwent nerve exploration for different reasons.

## Case Report

### Case 1

An 8-year old girl had fallen down from the monkey bar and sustained right elbow swelling and pain with limitation of elbow motion. On examination, the right elbow was swollen and tender with signs of Radial Nerve palsy: unable to extend the wrist joint and reduced sensation over the anatomical snuffbox. The radiograph showed fracture of the right supracondylar humerus (Gartland III) with the distal fragment displaced postero-medially (Fig. 1A). On the diagnosis of closed supracondylar fracture of the right humerus with Radial Nerve injury, we explored the nerve in the operation theatre before attempting closed reduction. We found the Radial Nerve severely stretched by the proximal fracture end and nearly lacerated (Fig. 1B), and was then released after fracture reduction. The fracture was held with crossed K-wires.

**Fig. 1a:**
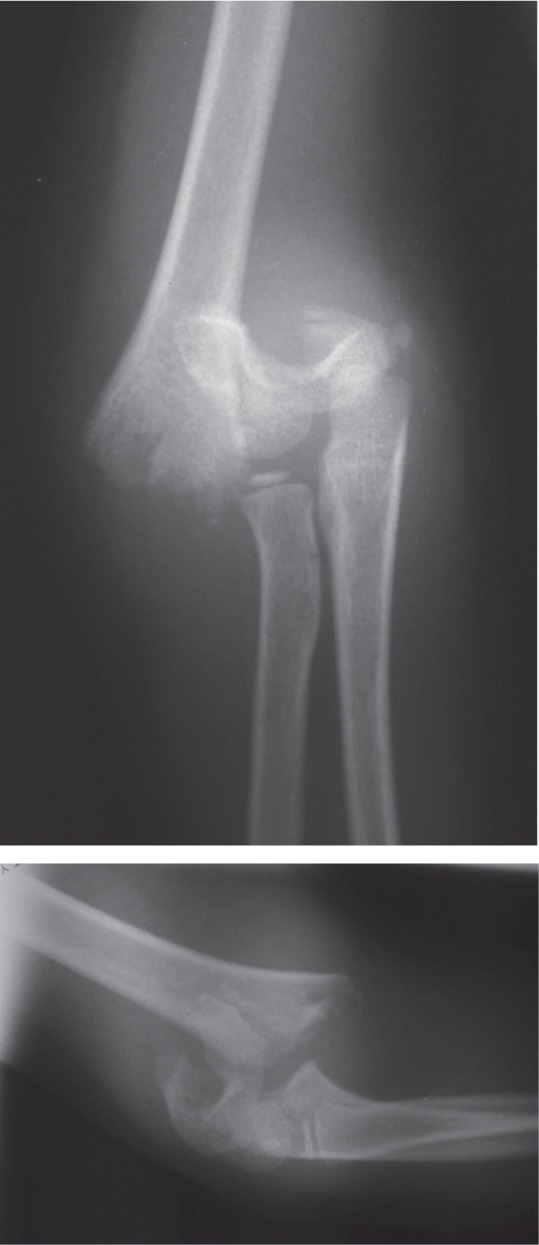
Radiograph showing fracture of the supracondyle of right humerus.

**Fig. 1b:**
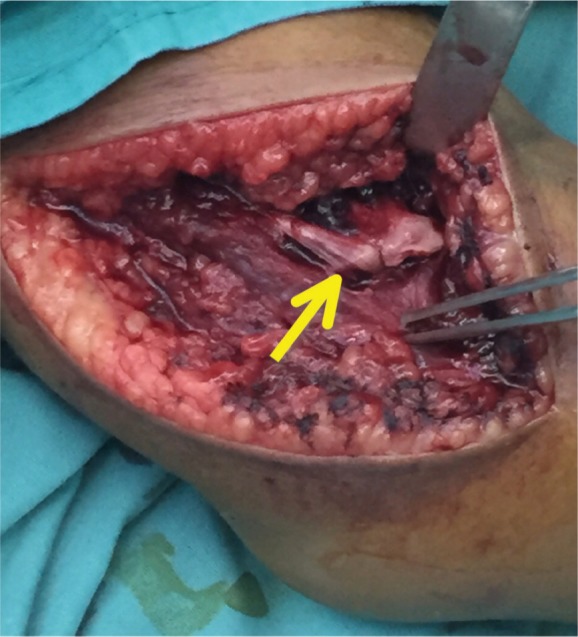
Radiograph showing fracture of the supracondyle of right humerus. Yellow arrow showing radial nerve.

### Case 2

A 5-year old girl had fallen on her out-stretch left hand while playing and sustained injury to her left elbow. On examination, the left elbow was tender and swollen with antecubital ecchymosis, with signs of Median Nerve injury: she was unable to flex the inter-phalangeal joint (IPJ) of the left thumb, distal inter-phalangeal joint (DIPJ) of the left index finger and reduced sensation over the Median Nerve distribution. Radiography showed left supracondylar humerus fracture (Gartland III) (Fig. 2A). As she had closed left humerus supracondylar fracture with Median Nerve injury, we attempted reduction under sedation in the casualty (emergency department) aiming to release the tension on the nerve. Immediately post-reduction the nerve had partially recovered with residual inability to flex the inter-phalangeal joint (IPJ) of the left thumb and reduced sensation over the Median Nerve distribution. On transferring her to the operation theatre, we attempted closed manipulative reduction (CMR) but failed, and proceeded with open reduction. We used this opportunity to explore the nerve and found the Median Nerve as well as the Anterior Interosseous Nerve (AIN) intact (Fig. 2B). The fracture was held with crossed K-wires.

**Fig. 2a:**
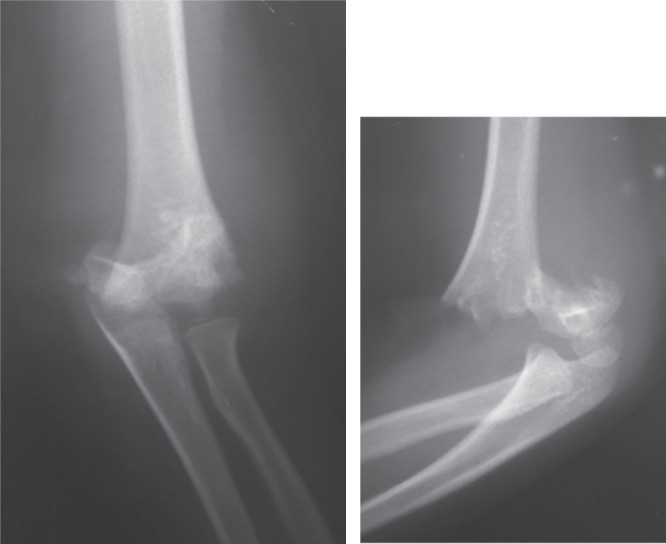
Radiographs showing fracture of the supracondyle of left humerus.

**Fig. 2b:**
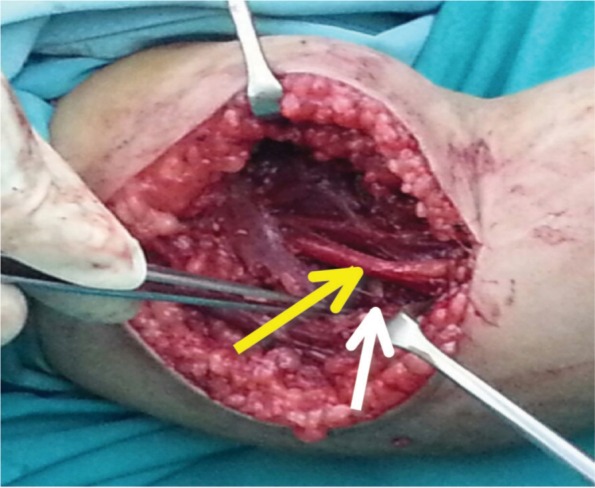
White arrow: Proximal fracture end. Yellow arrow indicates- intact Median Nerve.

### Case 3

A 10-year old boy had fallen on his out-stretch left hand and sustained left elbow pain and swelling. On examination, the left elbow was mildly swollen and tender without signs of neurological injury. The radiograph showed fracture of the left humerus supracondyle (Gartland III). On the diagnosis of displaced closed supracondylar fracture, we performed CMR and percutaneous pinning. The mini-open approach was used to place the medial wire (Fig. 3A). Immediate postoperatively, the patient developed signs of Ulnar Nerve palsy: mild claw hand with inability to flex the DIPJ of the little finger and reduced sensation over the Ulnar Nerve distribution. On suspicion of an iatrogenic Ulnar Nerve injury, we explored the nerve and found the medial wire impinging on the nerve (Fig. 3B) and after removal of the medial K-wire we noted an intra-neural blackish segment that could be intra-neural ischaemia or intra-neural hematoma (Fig 3C). A new medial wire was then inserted at a safe distance from the nerve.

**Fig. 3a:**
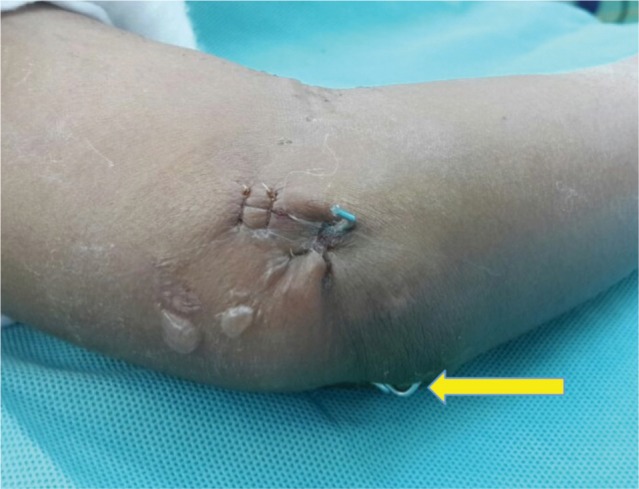
Mini-open wound with medial K-wire.

**Fig. 3b:**
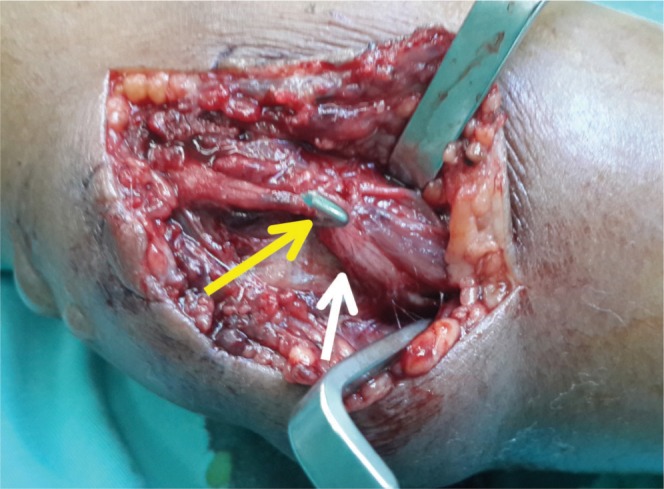
Medial wire (yellow arrow) was impinging the nerve (white arrow).

**Fig. 3c:**
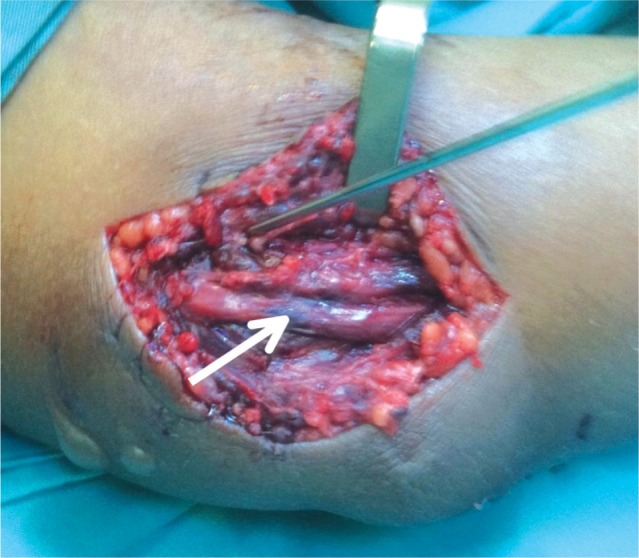
Intra-neural blackish segment that can be intra-neural “necrosis” or intra-neural hematoma (White arrow).

## Discussion

The issue of whether to explore or not to explore the injured nerve in SCHF is still controversial and a matter of continuing discussion among orthopaedic surgeons. Most of literatures report that the nerve may recover spontaneously over an average duration of 3-5 months or it may extend to as long as 7-8 months^4–5^. Thus, the majority of surgeons would choose to ‘wait and see’ the progress of the nerve healing and only plan for second surgery if there was no nerve evidence of recovery.

By exploring the nerve primarily, it is possible to confirm the nerve status, whether it is still in continuity or transected. If the nerve is found transected immediate end to end nerve repair can be done and this earlier repair gives assurance of a better prognosis. Furthermore, this acute nerve exploration at the same time as the fracture fixation avoids the need for subsequent surgery.

Up to the present time, there is no standard guideline for early management of nerve injury in SCHF. In Case 1 the Radial Nerve was severely stretched at the proximal fracture end and was released after reduction; therefore we would suggest that CMR should be performed as early as possible in the casualty department in SCHF with nerve palsy. The aim of the maneuver is not to achieve perfect reduction but only to adequately release nerve tension at the fracture site. Prolonged nerve stretching at the sharp fracture end may place the nerve at risk of nerve laceration or transection.

We performed immediate CMR for Case 2 at the casualty department and the injured Median Nerve had partially recovered before going to the operation theatre. As closed manipulation failed to reduce the fracture, we used this opportunity to open and explore the nerve. This would avoid subsequent surgery in the future to explore the nerve if the nerve had not recovered as we already know the nerve status from the initial surgery. Therefore we suggest -primary nerve exploration for any SCHF with nerve injury that requires open reduction.

The iatrogenic Ulnar Nerve injury can be due to traction -from multiple attempts at CMR in the operation theatre, medial pinning or ‘over-shoot’ of lateral wires. Majority of cases are due to the medial wire. As the Ulnar Nerve lies closely posterior to the medial epicondyle -it has greater risk to be penetrated, pierced or constricted by surrounding soft tissue while placing the medial wire^1^. The mini-open technique minimizes this injury. The management in iatrogenic Ulnar Nerve injury includes immediate nerve exploration, removal of the medial wire and ‘watch and see’^5^. The removal of medial wire may affect the stability of fracture fixation and the ‘watch and see’ is to await self-recovery of the nerve.

In Case 3, the initial plan was to only explore the Ulnar Nerve. If the nerve is intact and not related to the medial wire, we would just have closed the wound. If the nerve injury was related to the wire, we would revise the medial wire position. As we explored the nerve in this case, the wire was impinging on the nerve and the intra-neural blackish segment can be intra-neural ischaemia secondary to the nerve impingement. If the nerve exploration was not performed, the impingement would have persisted, subsequently causing permanent nerve damage. As the patient comes with intact neurology and the neurological deficit only appears after surgery, our role is to find the reasons and explain to the patient’s parents regarding the cause and prognosis.

In conclusion, from these three cases we recommend immediate CMR in the casualty in any case of SCHF with nerve injury and primary nerve exploration if it is decided to proceed with open reduction. For iatrogenic nerve injury, the nerve exploration gives more definite reason for the injury and explanation to the parents is important for them to foresee the potential nerve recovery in their child.
